# Targeting Oxidative Stress to Treat Vitiligo: Clinical and Molecular Evidence

**DOI:** 10.3390/biom16040612

**Published:** 2026-04-21

**Authors:** Noemi Aprile, Simona Scano, Barbara Bellei, Alberto Marini, Angela Filoni

**Affiliations:** 1 UOSVD Dermatology and Allergology, Hospital Vito Fazzi, 73100 Lecce, Italy; noemi.aprile07@hotmail.it; 2 Laboratory of Cutaneous Physiopathology and Integrated Center of Metabolomics Research, San Gallicano Dermatological Institute, IRCCS, 00144 Rome, Italy; simona.scano@ifo.it (S.S.); barbara.bellei@ifo.it (B.B.); alberto.marini@ifo.it (A.M.)

**Keywords:** oxidative stress, pigmentation, antioxidant, melanocytes

## Abstract

Vitiligo is a chronic autoimmune disease characterized by the destruction of epidermal melanocyte, resulting in well-demarcated white patches on the skin. Despite the established use of corticosteroids and calcineurin inhibitors and the recent introduction of Janus kinase (JAK) inhibitors, a breakthrough targeted therapy that interrupts the IFN-γ signaling pathway, stable repigmentation remains a major clinical challenge, necessitating deeper investigation into its pathogenesis. Among the factors contributing to vitiligo, including genetic predisposition and autoimmunity, oxidative stress is a central driver of melanocyte damage and the subsequent autoimmune response. Chronic oxidative disequilibrium (high ROS level and impaired mitochondrial activity) and reduced antioxidant capacity (Nrf2/ARE pathway and catalase deficiency) function as triggering factors upstream of most other pathogenic pathways. Consequently, targeting oxidative stress, either as a monotherapy or in synergy with emerging targeted treatments, remains a pivotal area of therapeutic interest even in the current era of targeted therapies. Still, a significant gap remains the lack of standardized oxidative biomarkers to monitor disease activity and therapeutic response. Identifying these indicators is essential for personalized clinical management in vitiligo. This review examines how chronic oxidative disequilibrium and a reduced antioxidant capacity initiate and sustain the autoimmune cascade, leading to disease onset and progression.

## 1. Introduction

Vitiligo is a chronic acquired autoimmune disorder characterized by the disappearance of melanocytes, the skin pigment-producing cells, leading to well-marked, oval, circular, or linear-shaped, pearly white macules and patches, varying from a few millimeters to centimeters in size and centrifugally expanded. It results from the combination of progressive melanocytes loss and the inadequate rescue of tissue integrity since spontaneous remission is extremely rare in skin vitiligo [1]. This latter aspect, associated with the concept of dysfunctional tissue regeneration or incomplete damage overcome, is largely underestimated. In line with the concept that white spot may represent a kind of “scar”, several additional alterations are present in vitiligo depigmented skin. Vitiligo skin exhibits impaired epidermal permeability barrier function, characterized by delayed barrier recovery after disruption, delayed reepithelization and lower erythema index compared to non-lesional skin [2,3]. Stratum corneum hydration is significantly reduced in involved skin compared to non-involved areas and keratinocytes differentiation is deregulated, leading to altered lipid composition, including imbalances in cholesterol, free fatty acids, and ceramides essential for barrier integrity [4].

Vitiligo can affect any area of the body, including the hands, face, hair, and genital region, significantly impacting patients’ quality of life due to remarkable cosmetic concerns [5]. The intensity of the disease is determined by the affected surface area of the body [6]. Vitiligo’s estimated prevalence ranges from 0.5% to 1–2%, equally affecting individuals across all age groups, regardless of sex, skin type, or ethnicity [6]. Vitiligo can be classified, according to Vitiligo Global Issues Consensus Conference (VGICC), as segmental (SV), where depigmented lesions only appear on one side of the body; non-segmental (NSV), that is further divided into generalized, acrofacial, universal, and mixed (associated with SV) types; and undetermined/unclassified vitiligo, including the focal and mucosal types [7]. The condition is often associated with other autoimmune diseases, including thyroid diseases, alopecia areata, and diabetes mellitus [8]. These diverse tissue-specific autoimmune conditions appear to share a predominant Th1-based immune response, characterized by elevated circulating levels of C-X-C motif chemokine ligand 10 (CXCL10) and a concomitant reduction in regulatory T cells (Tregs), suggesting an underlying unified pathogenic pathway [9]. The exact cause of immune system activation in vitiligo remains unknown but is believed to involve multiple factors, including genetic predisposition, metabolic alterations, defects in melanocyte adhesion to the epithelium, immune dysregulation, oxidative stress, and environmental triggers [10,11].

Oxidative stress plays a crucial role in the pathogenesis of vitiligo, particularly in genetically predisposed individuals. This excess of reactive oxygen species (ROS) accumulation initiates a cascade of events leading to chronic cellular damage and ultimately melanocyte death. Enhanced melanocyte precursor mobilization with possible premature stem cell exhaustion as well as the persistence of oxidative equilibrium in depigmented skin may be both implicated in reduced capacity to restore physiological pigmentation. This complex interplay between oxidative stress and immune activation is central to the progression of vitiligo. Oxidative modifications of cellular proteins may create neoantigens that the immune system no longer recognizes as “self.” This process, potentially exacerbated by the release of intracellular components upon melanocyte death, is thought to activate autoreactive T cells, establishing a self-perpetuating feedback loop where initial biochemical stress could transition into a systemic autoimmune attack.

Proving the centrality of oxidative equilibrium in vitiligo, the imbalance in the antioxidant system, the accumulation of ROS and marked sensibility to external oxidative injury has been proven in melanocytes located in the normally pigmented skin of vitiligo patients [12,13]. Furthermore, keratinocytes and fibroblasts also display imperfect management of oxidative stress [14], extending this disease-specific feature to the entire skin. Moreover, biochemical and functional alterations of vitiligo peripheral blood mononuclear cells (PBMCs) proved a systemic metabolic impairment [15,16], suggesting that vitiligo is not simply a skin disease.

This review focuses on oxidative stress in vitiligo, considering two different but interconnected aspects, the molecular pattern of redox disequilibrium and the clinical evidence of oxidative damage.

## 2. Concise Overview of Oxidative Stress

Oxidative stress occurs when production of pro-oxidant exceeds cellular antioxidant defenses, leading to disruption of redox signaling control and macromolecular damage. Chemically, oxidative stress is associated with elevation of free radicals, including O_2_^•−^ (superoxide radical), ^•^OH (hydroxyl radical), ^•^OH, ROO^•^ (peroxyl), ^•^NO (nitric oxide), and ^•^NO_2_ (nitrogen dioxide), and non-free radical H_2_O_2_ (hydrogen peroxide), which are normally present in cells at low level [17]. H_2_O_2_ is particularly unsafe because the lack of charge facilitates its diffusion through biological membranes, potentially triggering cellular harm.

ROS arise from both endogenous and exogenous sources. Endogenous ROS production occurs primarily in cellular compartments with high oxygen utilization, including mitochondria, peroxisomes, and the endoplasmic reticulum (ER), and to a lesser extent the cytosol and plasma membrane. Aging is another important intrinsic factor impacting on the loss of redox homeostasis [18]. On the other hand, several studies demonstrated that persistent oxidative stress accelerates senescence, affecting senescent-associated secretory phenotype (SASP) [19,20,21]. It has been estimated that mitochondria produce about 90% of intrinsic ROS [22]; thus, intense metabolic activity is constitutionally a source of oxidative stress. Thus, the overall redox equilibrium and the strategies adopted to preserve the homeostasis present peculiarity at cell type, tissue and organ levels. The melanin synthesis is a melanocyte lineage-specific additional source of ROS due to the conspicuous energy request associated to this biosynthetic process and the large amount of pro-oxidant intermediates generated [23]. Particularly, pheomelanin and its precursors are highly pro-oxidant compared to eumelanin [24]. However, the confinement of melanogenesis and pigment storage within specialized organelles named melanosomes, which are enriched in antioxidant enzymes [25], provides physiological protection to preserve cytoplasmic redox homeostasis. Consistent with the notion that melanogenesis requires an enhanced supply of antioxidants, catalase activity positively correlates with the degree of pigmentation and the expression of tyrosinase, the rate-limiting enzyme in melanogenesis [25,26].

Intracellularly, ROS impact on the modulation of several different signaling pathways such as mitogen-activated protein kinases (MAPKs), phosphoinositide 3-kinases (PI3K-Akt), phosphatase and TENsin homolog (PTEN), and nuclear factor kappa-light-chain-enhancer of activated B cells (NF-kB) [27]. Notably, ROS also promote antioxidant expression through activation of the transcription factor nuclear factor erythroid 2-related factor 2 (Nrf2). This factor binds to antioxidant response elements in promoter regions, thereby upregulating multiple genes involved in antioxidant defense and detoxification [28]. The redox system is also deeply implicated in metabolic pathway and ROS-mediated immunometabolism rearrangement is a crucial step for proliferation, survival and function of T cells, B cells and macrophages [29]. Accordingly, at the systemic level, ROS have a critical role in immune system function [30]. Oxidative stress overload is at the basis of the major tissue-specific autoimmune diseases, including vitiligo [31].

Exogenous contributors to oxidative stress include environmental factors such as ultraviolet (UV) radiation, pollutants, tobacco smoke, alcohol, toxins, and certain medications. The skin, as the organ most exposed to external insults, is particularly vulnerable to UV rays, which exacerbate oxidative stress both directly and indirectly by enhancing melanin synthesis and distribution as a protective mechanism against further sun exposure. The epidermis, the outermost cutaneous layer, resists exogenous insults primarily through physiological continuous turnover, which prevents the accumulation of excessive damage, as well as through a consistent regenerative capacity. Differently, melanocytes dispersed into the deepest epidermal basal layer are long-lived, slow-cycling cells prone to accumulating damage [32].

## 3. Genetic Predisposition, Oxidative-Stress-Related Genes

Much evidence highlights the importance of genetic background in the development of vitiligo. Important studies focused on European populations identified approximately 50 susceptible genes or genomic regions associated with vitiligo [33,34,35,36]. More recently, a relationship between polygenic risk burden and the age of onset in vitiligo has been identified, showing that individuals with higher genetic risk scores are more likely to develop the disease at an earlier age [37]. Vitiligo exhibits a robust genetic basis, as evidenced by genome-wide association studies that have pinpointed loci influencing oxidative stress response mechanisms, immune regulatory networks, and melanocyte-specific functions. Considering oxidative stress, emerging studies have focused the attention on genetic variants of antioxidants enzymes associated with vitiligo occurrence. Patients with vitiligo have not only significantly increased average levels of hydrogen peroxide in their skin lesions but also significantly lower activities of key antioxidant enzymes catalase (CAT), superoxide dismutase (SOD), glutathione peroxidase (GPX) and glutathione S-transferase (GST).

The origin of CAT deficiency in vitiligo patients has not been fully elucidated yet. Some studies reported a mechanism of substrate inhibition of catalase activity due to high levels of hydrogen peroxide from various sources in the epidermis of vitiligo patients [38,39]. A few allelic variants of the *CAT* gene associated to defective expression or function of the enzyme have been described for different diseases, e.g., hypertension, diabetes mellitus (type I and type II), insulin resistance, dyslipidemia, asthma, systemic lupus erythematosus, rheumatoid arthritis, malnutrition and vitiligo [40,41]. Casp et al. analyzed three CAT gene single-nucleotide polymorphisms (SNPs) in non-segmental vitiligo patients form North America, proving a significant association between the C/T SNP in codon 389 of exon 9 and the disease risk due to change in its expression in melanocytes and keratinocytes [42]. Another study focusing on the 5′-untraslated region of *CAT* gene revealed a 6.4-fold increased risk of vitiligo in individuals harboring the haplotype −89A/T and 20T/C, whereas no association was demonstrated for −262G/A [43]. SNPs of the *CAT* genes A-89T (rs7943316) and C419T (rs11032709) strongly associated to vitiligo in the Saudi population [44]. However, only subjects with susceptible genotypes/haplotype for CAT −89A/T and −20T/C polymorphisms showed significantly reduced CAT mRNA/activity [43]. However, other studies reported the opposite conclusion. In the Italian population restricted to CAT variant −89A/T a biological significance for vitiligo was found [45] and a study regarding Turkish vitiligo patients did not find significantly different frequency in this variant and 389C>T polymorphism compared to controls [46]. No association was found between the *CAT* −89A/T polymorphism and vitiligo in a Korean cohort [47], whereas Liu et al. (2010) reported that AT and TT genotypes were linked to elevated vitiligo risk in a Chinese population [48].

The relative risk for development of vitiligo was found as a 2-fold increase in the TT genotype of *SOD2* Ala9Val (C/T) SNP [49]. The Ala-9Val polymorphism has been extensively investigated in Parkinson’s disease and diabetes [50]. An interesting observation concerns the compromised transfer of SOD2 carrying homozygous TT into mitochondria [51]. Laddha et al. reported elevated SOD transcript levels in vitiligo patients, associated with SOD2 Thr58Ile (rs35289490) and Leu84Phe (rs11575993) polymorphisms, as well as the SOD3 Arg213Gly (rs8192291) polymorphism, whereas no genotype–phenotype correlation was observed for SOD1 expression levels [49]. Mechanistically, these genetic characteristics drive higher activity of SOD isoforms, which, if not coupled to an adequate catalase activity, led to cytoplasmic, mitochondrial, and extracellular accumulation of H_2_O_2_.

Considering the glutathione metabolism, genetic variants of both GST and GPX are correlated with vitiligo. Mansuri et al. highlight the key role of less functional glutathione metabolism gene variants in vitiligo susceptibility [52]. They found an increased frequency of GPX1 polymorphisms Arg5Pro and Leu6Pro in vitiligo patients versus controls [52]. The Arg-to-Pro substitution reduced GPX1 activity and increased lipid peroxidation in erythrocytes, despite no link to lower GPX1 transcript levels [52]. Studies have highlighted the significant association between *GSTM1* and *GSTT1* null polymorphisms and the risk of vitiligo onset, since homozygosity for the null allele of the GSTM1 and GSTT1 results in the lack of expression of the enzyme [53,54,55]. The null genotype of GSMT1 and GSTT1 has been linked to vitiligo in Korean [56] and Chinese [57] populations, respectively. Guarnieri et al. found a higher frequency of the double null genotype GSMT1null/GSTT1null in vitiligo patients versus the double-active genotype [55] in a Mediterranean population, a trend also seen in Egyptian patients [58,59]. Differences among studies may result from variations in genetic background and environmental factors affecting the overall antioxidant response.

The aryl hydrocarbon receptor (AhR) is a ligand-activated transcription factor that regulates gene expression involved in cell differentiation and immune response. AhR activation promotes antioxidant pathways, suppresses abnormal immune responses, and upregulates melanogenesis genes, protecting melanocytes from oxidative stress, controlling disease progression, and aiding lesion repigmentation. Studies show abnormal AhR expression in vitiligo, with decreased levels in lesional skin [60] and peripheral blood mononuclear cells linked to disease severity [61]. Decreased expression of the AhR receptor is associated with elevated IFN-γ production and diminished upregulation of immune checkpoints in vitiligo [62]. Because activation of AhR pathway helps to activate antioxidant pathways, inhibit abnormal immunity response, and upregulate the melanogenesis gene, thereby protecting melanocytes from oxidative stress damage, the therapeutic usage of AhR agonist has been recently proposed [63]. The only reported *AHR* polymorphism is the protective T allele of −129C/T located in the promoter region [64]. Notably, −129T allele is associated with elevated expression of *AHR* mRNA and, in vitiligo, it associated to higher TNF-α concentration and decreased levels of IL-10 and TGF-β1 in the serum of vitiligo patients compared with controls [61]. AhR directly modulates the Nrf2 pathway, increasing antioxidative enzymes like heme oxygenase-1 (HO-1) and NAD(P)H:quinone oxidoreductase 1 (NQO1), vital for melanocyte redox homeostasis [65]. It has been shown that the *NR2* rs35652124 T/C polymorphism and serum HO-1 activity affect susceptibility to vitiligo among the Han Chinese population [66]. Furthermore, Sorour et al. demonstrated that *Nrf2* −617 T/G and −653 T/C polymorphisms might play a role in patients’ susceptibility to vitiligo and modify the clinical presentation of the disease [67].

A recent Turkish study linked human leukocyte antigen (HLA) polymorphisms to antioxidant capacity, underscoring the interplay between immune-mediated melanocyte destruction and oxidative stress [68]. Vitiligo patients showed higher HLA-A*02* allele frequency, especially in late-onset cases, but lower HLA-A11 and HLA-DRB101 frequencies, indicating a protective effect. Although serum total antioxidant capacity (TAC) was comparable between patients and controls, it was reduced in those lacking HLA-DRB101, which was more prevalent in controls and high-TAC patients, suggesting an HLA-TAC protective interaction [68]. Thus, it is tempting to hypothesize that the mixture of extracellular vesicles, cellular fragments, and protein aggregates from damaged skin cellular populations could boost the T cell activation and response, supporting the autoimmunity process in vitiligo [69,70,71].

## 4. Oxidative Stress in Vitiligo: Systemic Markers

Studies demonstrated that specific serum indicators of oxidative stress, including total antioxidant capacity (TAC), malondialdehyde (MDA), and 8-hydroxy-2′-deoxyguanosine (8-OHdG), could effectively reflect disease activity and severity in patients with vitiligo [72]. The literature shows conflicting data on oxidant and antioxidant levels, including SOD, in vitiligo patients. Some groups reported higher SOD activity in the blood of patients [73], whereas others described a reduction [72,74,75,76]. Most of these investigations demonstrate reduced catalase activity in the blood of vitiligo patients compared to controls. In the wake of this [16] have shown that SOD activity and MDA levels were significantly higher in red blood cells of vitiligo patients, positivity correlated with disease extent and activity, while catalase and G6PD activities were significantly lower. This systemic antioxidant imbalance supports the hypothesis that widespread enzymatic deficiencies underpin vitiligo pathogenesis.

## 5. The Pathogenic Picture of Vitiligo Collocates Intrinsic Oxidative Disequilibrium in the Joint of Melanocyte Destruction Network

**Figure 1:**
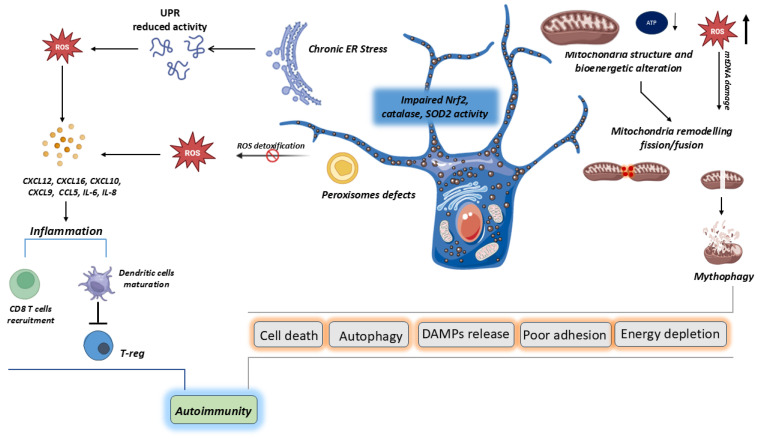
Oxidative stress in vitiligo.

The pathogenic profile of vitiligo reveals an intrinsic oxidative disequilibrium deeply embedded within the melanocyte destruction network. This imbalance generates a chronic proinflammatory milieu, which triggers autoimmune mechanisms amplifying this process, targeting melanocytes through T-cell-mediated cytotoxicity and cytokine storms. Several endogenous interconnected cellular activities trigger oxidative stress (Figure 1).

In vitiligo, alterations in the mitochondrial structure and activity of melanocytes have been observed, associated with reduced ATP production and increased oxidative stress, which leads to enhanced mitochondrial dynamics. The chronic accumulation of reactive oxygen species (ROS) in the endoplasmic reticulum (ER) generates a buildup of misfolded proteins that initiate the Unfolded Protein Response (UPR). Due to functional defects, the UPR further exacerbates oxidative stress, triggering the production of proinflammatory cytokines and chemokines. These factors contribute to the recruitment of T lymphocytes and the maturation of dendritic cells. Additionally, defects in peroxisomes prevent proper detoxification, further amplifying the inflammatory process.

### 5.1. Mitochondria, the Metabolic Hub

Mitochondria are the powerhouse of intracellular metabolism, generating adenosine triphosphate (ATP) through oxidative phosphorylation (OXPHOS). Glycolysis contributes to ATP production through an anaerobic catabolism of glucose, which, occurring in the cytoplasm, outside the mitochondria, releases two molecules of ATP. ATP provides energy to support most biological processes in living cells. At the cellular level, several mitochondrial abnormalities have been observed in vitiligo, including disruptions in mitochondrial structure and function. These include loss of the transmembrane potential [77,78], dysregulation of cardiolipin and proteins involved in the electron transport chain [79], accumulation of cholesterol in the inner mitochondrial membrane, and an increase in overall mitochondrial mass [15,79]. Aberrant mitochondrial activity impairs cellular bioenergetics, leading to attenuated ATP production and heightened metabolic/oxidative stress. Whereas vitiligo melanocytes and fibroblasts present a depolarization of the mitochondria membrane, an unexpected hyperpolarization characterizes vitiligo keratinocytes [78]. Previous studies have documented correlations between elevated intracellular ROS levels, reduced ATP production, and mitochondrial hyperpolarization in other chronic inflammatory conditions, such as systemic lupus erythematosus, where these changes contribute to T-cell hyperactivation [80]. Interestingly, more polarized mitochondria, sometimes associated to minor mitochondria mass, has been reported as a peculiar bioenergetic alteration in immune cells of type I [81] and type II [82] diabetes patients, reinforcing the association between vitiligo and metabolic illness.

Oxidative disequilibrium in vitiligo is likely exacerbated by metabolic activities but it is basically due to intrinsic factors since ROS are still abnormally elevated when metabolic activities were minimized by growth factors starvation of cell cultures [78]. Parallel, keeping vitiligo cells metabolically quiescent by starvation realigned the level of intracellular ATP to those of control samples [78].

Overall, these data strengthen the assumption that oxidative stress originates intrinsically whereas energetic defects emerge in the function of the metabolic requests. Insufficient ATP availability is an energy-deprived condition resembling hypoglycemia, a condition that promotes glucose import. Accordingly, in vitiligo, a compensatory glucose overload aims to rescue energy production, causing deregulated production of advanced glycation products (AGEs), non-enzymatically formed compounds implicated in oxidative stress and sterile inflammation. To attempt energy demand, mitochondria reprogram metabolism-related signaling pathways, such as AMP-activated protein kinase (AMPK)/mammalian target of rapamycin (mTOR) and peroxisome proliferator-activated receptor gamma coactivator 1-alpha (PGC-1α). In vitiligo cells, persistent perception of energy shortage leads to chronic mobilization of autophagic catabolic processes, lack of coordination between AMPK and mTOR/S6 signaling, favoring insulin resistance [78].

Imperfect mitochondrial activity gives rise to intensification of organelles remodeling. Dynamic shaping of mitochondria is a mechanism of quantity and quality control of these organelles and includes fission/fusion processes as well as mitophagy (a selective form of autophagy that specifically targets damaged or dysfunctional mitochondria), a specific type of autophagy. Defective SIRT3 expression and activity in vitiligo melanocytes increase the rate of mitochondrial fission, cytochrome c release and apoptosis [83]. Although several researchers evidenced overactive autophagy [84,85], mitophagy seems to not be upregulated, a fact that could favor the accumulation of damaged mitochondria and subsequent destruction of melanocytes under oxidative stress [86].

### 5.2. Endoplasmic Reticulum’s Stress and Unfolded Protein Response in Vitiligo

ROS induce oxidative damage to the whole range of cellular macromolecules, including proteins. When damaged and improperly folded proteins accumulate, cells initiate a pro-survival cascade named unfolded protein response (UPR), a mechanism aimed to restore the endoplasmic reticulum’s (ER) protein-folding homeostasis by reducing the global protein load and increasing the ER’s capacity to handle folding. If the stress is too severe, the UPR can lead to the inhibition of protein synthesis to prevent translational burden to the ER and eventually culminate with the arrest of cell cycle or cell death to protect the entire organism [87]. The UPR regulates protein homeostasis and inflammation through the PERK/eIF2α and ATF6 pathways [88,89], while the parallel IRE1α/XBP1 axis promotes proinflammatory cytokine expression and dendritic cell survival [90,91]. Together, these mechanisms highlight the UPR’s critical role in bridging cellular stress with immune response regulation. UPR is also a crucial regulator of nuclear factor kappa B (NF-kB) and activator protein 1 (AP-1) [92]. As a matter of fact, defective UPR is involved in the development of autoimmunity in various diseases [93,94], including type 1 diabetes mellitus [95], rheumatoid arthritis [96], Alzheimer’s disease [97] and vitiligo [98]. A specific peculiar relevance of UPR mechanism in the melanocyte lineage was evidenced by the attenuated response to chemical inducer of ER stress in tyrosinase-mutant melanocytes [98]. However, the significance of these data for vitiligo pathogenesis remains unclear, as chronic ER stress can attenuate the UPR even when tyrosinase accumulates significantly. Recently, Yamazaki et al. demonstrated that melanogenesis per se activates the UPR in B16 mouse melanoma cells and that its attenuation exerts a negative effect on melanin synthesis [99]. Additional players of the pigmentary system implicate UPR system in a cell-lineage-specific toxicity. Damaged gp100, a melanosome-specific matrix protein, and accumulated amyloid oligomers of this protein under ER stress are toxic and possibly contribute to vitiligo pathogenesis [98]. This aspect underlies the similarity with neurodegenerative diseases, since accumulation of unnecessary amyloid fibrils is typical of Parkinson’s and Alzheimer’s diseases [100]. Melanocytes from pigmented skin of vitiligo patients have dilatated ER, a typical feature of ER stress [101]. On the other hand, accumulation of misfolded proteins in the ER further augments oxidative stress [102]. When exposed on the cell surface or released in the extracellular space, misfolded proteins can function as neo-antigens, triggering autoimmunity. UPR activation in melanocytes exposed to exogenous stressors induces expression of interleukin (IL)-6 and IL-8 [103], two proinflammatory cytokines present at elevated levels in vitiliginous skin and/or serum [104,105]. Interestingly, IL-6 and IL-8 expression is partly regulated by XBP1 [106]. Additionally, polymorphisms in the promoter of XBP1 and associated to its expression modulation have been significantly correlated to the risk of developing vitiligo [107]. UPR activation promotes T-cell homing to depigmented areas by increasing CXCL12 and CCL5 in melanocytes [108] and CXCL16 in keratinocytes [109]. This sustained response fosters a proinflammatory environment essential to vitiligo pathogenesis. Additionally, the ER-to-membrane translocation of calreticulin, a damage-associated molecular pattern, is overexpressed in perilesional skin and correlates with disease severity [110]. Calreticulin stimulates the migration of CD8+ T-cell and the maturation of DCs, suggesting another link between oxidative stress and autoimmunity in vitiligo.

### 5.3. Peroxisomes: ROS Detoxification and Lipid Metabolism

Peroxisomes are small, membrane-enclosed organelles enriched in enzymes implicated in a variety of metabolism-related pathways. The oxidation of fatty acids, which provides a major source of metabolic energy in animal cells, occurs in both peroxisomes and mitochondria. Oxidative reactions into peroxisome are balanced by a high amount of catalase, which manages hydrogen peroxide degradation either by converting it to water or by oxidation of another organic compound. Scavenger activities include also the activation of PEX2 inhibiting lipolysis to counteract excessive accumulation of fatty acids and ROS [111]. Peroxisomal defects hinder ROS detoxification and fuel inflammatory cascades contributing to oxidative stress and possibly to melanocytes destruction in vitiligo. Recent research in human cell models indicates that peroxisomes can form physical contacts with mitochondria, facilitating the transfer of ROS to help maintain mitochondrial health [112]. Peroxisome–mitochondria interplay, where peroxisomal ROS overload sensitizes melanocytes to apoptosis, underscores a role in vitiligo pathogenesis. Subcellular organelles are highly interconnected, a fact that led to delocalization of melanosome-associated antigens in other intracellular structures in addition to lysosomes that are the melanosome precursors [113].

## 6. Molecular Evidence Supporting the Connection Between Oxidative Stress and Melanocytes Disappearance

These mitochondrial dysfunctions contribute to melanocyte damage and death, playing a significant role in vitiligo pathogenesis. Melanogenesis, the metabolic process responsible for pigment synthesis, is a highly demanding process that requires the optimal function of mitochondria. Thus, mitochondria dysfunction impacts melanogenesis, either promoting or inhibiting this complex process [114].

### 6.1. Melanocytes Adhesion

Oxidative stress impairs vitiligo melanocyte adhesion by inducing protein oxidation and lipid peroxidation, which disrupt key adhesion molecules like β1-integrin, E-cadherin, and laminin in the basal epidermis. Elevated ROS levels, particularly at lesion borders, weaken melanocyte–keratinocyte interactions and fibronectin binding, promoting detachment and transepidermal migration (melanocytorrhagy). This vulnerability sensitizes cells to anoikis and apoptosis, accelerating pigment loss.

Moreover, loss of melanocyte adhesion to the basal layer eliminates fibronectin-dependent inhibition of apoptosis, driving their upward migration through the epidermis and reducing viability. Accordingly, melanocytes in unstable vitiligo show diminished binding to type IV collagen, coupled with caspase-3 activation and heightened Annexin V expression, in contrast to the strong adherence observed in stable vitiligo and healthy controls [115,116].

### 6.2. Cell Death

Vitiligo melanocytes exhibit heightened susceptibility to multiple regulated cell death pathways, including apoptosis, necroptosis, pyroptosis, ferroptosis, parthanatos, and dysregulated autophagy, converging to accelerate pigment cell loss and stem cell exhaustion [117,118]. At the same time and because of cell death the release of immunogenic cell debris could occur in the skin of vitiligo patients a fact that might trigger immune response.

Enduring oxidative stress, mitochondrial dysfunction, and ER stress primarily trigger intrinsic apoptosis via Bcl-2/Bax imbalance, cytochrome c release, caspase activation, and TRPM2-mediated calcium dysregulation. Elevated levels of TRPM2 expression in vitiligo skin suggests this mechanism as a possible facilitator of vitiligo melanocytes loss [119]. Extrinsic apoptosis arises from T-cell-derived cytokines (IFN-γ, TNF-α, and FasL) and defective PD-L1 expression, compounded by adhesion defects promoting anoikis and melanocytorrhagy. In apoptosis, cytokine release remains minimal, whereas necroptosis triggers substantial secretion, fueling intense inflammation through IL-1β maturation and assembly of the NLRP3 inflammasome complex.

Necroptosis, pyroptosis, ferroptosis and parthanatos are lytic caspase-driven death with specific immunogenic dimensions [118]. Unlike the apoptotic process that is associated to package of cellular contents into sealed apoptotic bodies rapidly removed by neighboring cells, the type of cell death characterized by massive plasma membrane rupture triggers potent inflammatory responses and massive release of DAMPs like HMGB1, ATP, and calreticulin. Necroptosis involves RIPK3/MLKL phosphorylation leading to pore formation and NLRP3 inflammasome activation with IL-1β secretion. Pyroptosis and ferroptosis amplify sterile inflammation via gasdermin pores, lipid peroxidation, and alarmin exposure, contrasting non-immunogenic apoptosis. Parthanatos involves PARP1 hyperactivation from DNA damage, and impaired mitophagy, due to p53 elevation, sustains dysfunctional mitochondria, fueling ROS and apoptosis.

### 6.3. Premature Cell Senescence

Oxidative stress has additional consequences beyond cell death. ROS induce senescence.

In vitiligo pathogenesis, senescence has been identified as an important piece of the puzzle [13]. The persistent oxidative damage triggers signaling pathways that prevent cell division and accelerates cellular senescence. Senescent-like features have been demonstrated in melanocytes, keratinocytes, and fibroblasts coupled with the corresponding secretory activity named senescence-associated secretory phenotype (SASP) [120,121,122] consisting of a mix of cytokines, growth factors, and extracellular matrix metalloproteinases.

### 6.4. Immune Activation

Therefore, as hydrogen peroxide accumulates, melanocyte activates defense and self-protection systems such as HO-1 and the Nrf2-ARE pathway and autophagy. In vitiligo, however, these antioxidant responses are often inherently impaired and insufficient to restore redox homeostasis. Rather than operating in isolation, these pathways are deeply interconnected; their failure triggers the release of alarmin molecules, initiating a complex crosstalk with the innate immune system. This involves the activation of NLRP1 in dendritic cells, priming a proinflammatory microenvironment that orchestrates the recruitment of pathogenic CD8+ T cells [123]. As a highly conserved, oxidative-stress-sensitive transcription factor, hypoxia-inducible factor-1α (HIF-1α) regulates cytokine production and immune responses. HIF-1α levels are elevated in both the serum and depigmented skin of vitiligo patients [124]. Stressed keratinocytes upregulate this transcription factor, which in turn enhances the secretion of CXCL9 and CXCL10 chemokines, driving the local recruitment of lymphocytes [124]. IFN-γ is a central cytokine in the pathogenesis of vitiligo and can be defined as the “master player” of inflammation. IFN-γ levels have been shown to increase in the active phases of vitiligo and are closely correlated with the progression of the clinical picture. It acts directly on melanocytes, inhibiting melanin production, increasing oxidative stress, and promoting senescence and cell death [125]. These effects are primarily mediated by the JAK/STAT signaling pathway, which is overactivated in lesional skin [126,127]. In addition to direct damage, it stimulates the production of chemokines such as CXCL10, which attract autoreactive cytotoxic T lymphocytes to the skin [128]. Confirming the close relationship between immunological aspects and oxidative stress in vitiligo, a recent study by Veerabomma and collaborators reported that the therapeutic efficacy of an optimized topical formulation of the JAK inhibitor Tofacitinib was associated with oxidative stress mitigation [129]. IL-15 potentiates JAK-STAT signaling pathways in immune cells, with important implications in the homeostasis and activation of immune response [130]. IL-15 levels are elevated in vitiligo perilesional skin compared to healthy controls. Moreover, the quantity of IL-15 mRNA strongly correlated with H_2_O_2_ buildup, highlighting the link between oxidative stress and IL-15 expression. Consistently, the blockage of IL-15–JAK-STAT axis prevents oxidative-stress-induced CD8^+^T_EMs_ activation in vitiligo [131]. TNF-α levels are also increased in active vitiligo lesions. This cytokine impairs melanocytes by interfering with key regulators of melanogenesis, including MITF and the melanocyte-stimulating hormone receptor (MSH-R). It reduces the expression of the MC1-R receptor and exacerbates oxidative stress [132]. At the same time, however, TNF-α also appears to exert a regulatory function, promoting the expansion and activation of regulatory T cells (Treg). IL-33 appears to be a “double-sided” molecule, involved in both melanocyte damage and inflammation control mechanisms [133]. IL-33 is released by damaged keratinocytes and, once activated, inhibits melanocyte growth and amplifies inflammation by increasing the production of IL-6 and TNFα. It acts as an alarm signal. Elevated in vitiligo patients, its levels suggest a role in active disease phases. ROS accumulation triggers the release of melanocyte autoantigens, activating dendritic cells and macrophages. This innate response stimulates cytotoxic T lymphocytes (CTLs), leading to targeted melanocyte destruction and sustained inflammation [134,135,136,137].

Another key axis is the inflammasome, specifically NLRP1 and NLRP3, which leads to the production of IL-1β. This cytokine is closely associated with progressive vitiligo; it reduces the expression of MITF and is strongly correlated with disease activity and severity [138]. Oxidative stress further enhances IL-1 activation, promoting a persistent immune response. This cytokine is closely associated with progressive vitiligo; it reduces the expression of MITF. IL-6, produced by T lymphocytes and macrophages, is often increased, especially in the early or active phases of vitiligo [105]. Its expression is strongly influenced by oxidative stress and may contribute to the activation of the immune response following melanocyte damage [139]. Overactivation of the IL-6 autocrine signaling pathway, which acts in synergy with IFN-γ, exacerbates fibroblast senescence and drives melanocyte apoptosis [140]. This mechanism has been also implicated to explain psychological triggering and the link with psychological comorbidity [140]. Th17 axis cytokines, particularly IL-17, IL-21, and IL-23, are increased in many patients and are generally associated with the duration and extent of the disease. While not the primary driver of vitiligo, IL-17 inhibits melanogenesis and aggravates mitochondrial dysfunction and ROS accumulation [141].

Another cytoprotective mechanism against damage from different kinds of stress, including oxidative stress and incorrect folding linked to progressive loss of pigmentation, is heat shock protein (HSP). The Keap1/Nrf2 pathway enhances Hsp70 transcription, while ROS-induced oxidation of its cysteine residues modulates chaperone activity and redox signaling [142]. In melanocytes, Hsp70 specifically assists in the folding and transport of melanosomal proteins [143]. Notably, the inducible form, HSP70i, is overexpressed in vitiligo lesions and experimental models [144,145], with elevated mRNA in patient biopsies. HSP70i enhances DC/CTL activation by presenting melanocyte antigens on major histocompatibility complex (MHC) [146,147], amplifies IFN-γ/IFN-α signaling, and induces CXCL9/10 chemokines from keratinocytes to recruit CXCR3+ CD8+ T cells to lesional skin [148].

Overall, these findings highlight oxidative stress as a pivotal initiator of autoimmunity, driving early cell damage, antigen presentation, and CTL recruitment to depigmentation sites. Notably, vitiligo patients exhibit reduced regulatory T cells (T_reg_), which are essential for self-tolerance. Single-cell RNA sequencing suggests that Treg activity is modulated by the CCL5/CCR5 axis. Since CCL5 is regulated by UPR activation, oxidative imbalance likely dysregulates this signaling, further impairing immunotolerance in vitiliginous skin [108,149].

Table 1 summarizes the key molecular evidence and biomarkers supporting the role of oxidative stress in the onset and progression of vitiligo.

## 7. Therapeutic Interventions to Correct Oxidative Disequilibrium in Vitiligo

### Dietary Supplementation

The skin contains several natural antioxidants. These natural antioxidants include vitamin E, vitamin C, uric acid, squalene, and coenzyme Q10. Vitiligo patients are more affected by oxidative stress, implying inadequate protection by both enzymatic and small molecule scavengers. Consequently, while results regarding specific molecules vary, there is a consensus across studies that the total antioxidant capacity in the blood of vitiligo patients is significantly decreased. Therefore, several clinical trials have aimed to improve this capacity through the administration of the main enzymes/molecules involved in the oxidative process [150,151,152,153,154,155,156].

However, antioxidants as monotherapy yield very little results in skin repigmentation and clinical trial studies offer contradictory results [157,158]. Therefore, these tools are indicated to enhance repigmentation of standard pharmacology or phototherapy [157]. The use of vitamin E and C in combination with other therapies [159,160,161] provides great stabilizing effects to the cell membranes and neutralizes free radicals, particularly in young patients and patients with recent vitiligo onset [162]. Trace elements such as zinc, copper, and selenium also play an important part in maintaining cellular balance. Zinc and copper support key antioxidant enzymes and contribute to melanin synthesis [153,163], while selenium, essential for glutathione peroxidase, helps counteract oxidative stress [162]. What stands out is that both a deficiency and an excess of these elements can tip this delicate balance, highlighting the need for individualized supplementation [164,165]. Omega-3 fatty acids and alpha-lipoic acid (ALA) have attracted growing interest for their strong antioxidant and anti-inflammatory effects. When combined with narrow-band UVB therapy, both ALA and polyunsaturated fatty acids have shown the ability to enhance pigment restoration and protect melanocytes from damage [159,166,167].

New research avenues are also emerging from less obvious directions. The gut–skin axis, for example, is gaining considerable attention in vitiligo studies. Alterations in the gut microbiota may influence immune responses and oxidative stress at the skin level. Early evidence suggests that certain probiotics, such as *Lactobacillus plantarum*, may help reduce inflammatory cytokines and improve the body’s overall antioxidant defenses [168]. A recent meta-analysis showed that *L. plantarum* can increase IL-10 levels while lowering IL-4, IFN-γ, and TNF-α, suggesting a potential role in balancing pro- and anti-inflammatory pathways [169]. While these findings are promising, longer and more robust studies are still needed to confirm the therapeutic value of this approach in vitiligo. Interest in complementary and alternative medicine, including herbal remedies, has also grown in recent years. Plant-derived compounds are increasingly being explored as potential adjuncts to clinical therapies. Among these, *Ginkgo biloba*, a traditional Chinese herb rich in polyphenols and flavonoids, stands out for its potent antioxidant and immunomodulatory properties [162,170]. Its ability to reduce the activity of cyclooxygenase and TNF-α helps dampen inflammation and counter oxidative damage, both central to the disease process [171,172]. Also, *Phyllanthus emblica* has a high antioxidant capacity due to its rich polyphenolic and vitamin C content. Its fruit extract effectively inhibits lipid peroxidation and scavenges diverse free radicals, including superoxide anions and nitric oxide [160]. Similarly, clinical trials involving *Polypodium leucotomos*, a tropical fern extract, found that it enhances the results of UVB phototherapy while simultaneously protecting skin cells from oxidative injury [173,174]. Thymoquinone, the primary component found in *Nigella sativa* seeds, shields organs from oxidative harm, in fact its application improves significant repigmentation in the hands, face and the genital region of vitiligo patients [175]. Quercetin (QUE) is anti-apoptotic and antioxidant and works along with protecting ER function, reducing ROS, and synergizing with luteolin and kaempferol in Baishi Tablets type formulations to survive melanocytes 0 [176]. Curcumin activates the Nrf2 pathway and affects proinflammatory factors to cell protection from oxidative damage and possible enhancement of repigmentation along with phototherapy. In vitiligo, while in vitro experimental studies encouraged its use to protect vitiligo skin [177,178], only one randomized patient-based study reported enhanced efficacy of phototherapy when associated to topical tetrahydrocurcuminoid cream administration [179]. KF in kaempferol-3-O-glucoramnoside bypasses the triad of autosomal recessive in melanocytes destruction by advanced immune systems [180]. Glycyrrhizin further complements these mechanisms by protecting melanocytes through Nrf2-dependent HO-1 induction and exerting immunomodulatory and anti-inflammatory effects, though its use requires caution in patients with hypertension or cardiovascular disorders. More comprehensive clinical studies are essential, with rigorous clinical validation, to guarantee safety effectiveness of these compounds [181].

Table 2 summarizes the clinical trial data on therapeutic agents targeting oxidative stress.

## 8. Pharmacological Treatments to Counteract Oxidative Stress

In recent years, several preliminary studies have begun to evaluate the potential of afamelanotide, in combination with phototherapy, in promoting repigmentation of non-segmental vitiligo. Open studies have demonstrated tolerability and efficacy, showing that the most common side effect is hyperpigmentation with occasional headache, nausea and dizziness [182].

## 9. Regenerative Medicine-Based Therapeutic Strategy with Implication in Redox Homeostasis

Regenerative medicine strategies are primarily founded on two biological principles: the enhancement of the tissue’s innate reparative potential and the direct restoration of the cell population through transplantation. Due to the limited efficacy of conventional treatments, recent years have seen the proposal of cell-based and acellular interventional therapeutic strategies to achieve more effective repigmentation. Several of these approaches have provided clinical and preclinical evidence of desirable effect in counteracting oxidative stress.

A major interest in regenerative medicine applied to vitiligo consists of replacing pigment cells in the lesional areas via autologous grafting. Cellular therapies, including non-cultured epidermal cell suspension, cultured melanocytes, non-cultured follicular root sheath suspension graft, mesenchymal stem cells (MSC) or multilineage-differentiating stress enduring (MUSE) cell-based therapies, alongside tissue graft (such as epidermal sheet transplantation, mini-punch graft, split-thickness skin graft and hair follicle graft) offer various possibilities for replacing missing cells [183]. However, in principle, a hostile microenvironment may prevent the survival of transplanted material and reduce treatment effectiveness. Lin and collaborators identified the presence of inflammatory markers as a negative predictor of cultured melanocyte transplantation [184]. A similar scenario is plausible also for the redox background. Consequently, grafting techniques employed in vitiligo treatment may be optimized through the adjunctive use of antioxidant-rich formulations to treat the local tissue site and enhance clinical success. The use of adipose-derived stem cells (ADSCs) has gained attention due to their multi-lineage differentiation potential and the antioxidant and anti-inflammatory feature. When cultured with specific trophic factors, these cells undergo a morphological shift toward bipolar or dendritic shapes characteristic of mature melanocytes [185]. This transition is marked by the progressive expression of essential melanogenic differentiation markers, including Mitf, Tyrosinase, Trp1, and Trp2 [185,186]. Given that persistent oxidative imbalance is fundamental to the development of vitiligo, leveraging the ROS-neutralizing capabilities of ADSCs offers a promising therapeutic avenue to mitigate cellular damage. Preliminary in vitro evidence demonstrated that the addition of mesenchymal stem cells to melanocyte cultures treated with H_2_O_2_ supports protective activities [187]. Focusing on potential acellular treatments, ADSC-derived secretomes exhibit significant antioxidant potential by upregulating the expression and enzymatic function of key protective molecules, such as SODs, GPx, catalase, and HO-1, within epidermal and dermal cells, protecting them from UV and oxidative damage [188,189]. Furthermore, the co-transplantation of melanocytes with MSCs enhances pigment cell proliferation [190] and migration [191]. Supporting the idea that MSCs may act as a functional scaffold for vitiligo melanocytes grafts, ADSCs have been shown to improve the efficacy of melanocytes transplantation in two animal models [192,193].

The application of platelet-rich plasma (PRP) in vitiligo patients stems from the concept that persistent white patches resemble a “wound that never heals”. PRP has been reported as a potential treatment for vitiligo by promoting melanocyte regeneration, strengthening intercellular adhesion, and providing anti-inflammatory effects [194]. PRP is rich in melanocyte-supportive growth factors such as bFGF, SCF, IGF-1 and TGFβ, which are essential for melanocyte function [195,196]. More recently, studies focused on the therapeutic use of exosomes derived from platelet-rich plasma (PRP-exo). The restoration of redox equilibrium by this type of preparation promoted hair regeneration in mice [197] and accelerated diabetic wound healing [198] by alleviating oxidative stress. Several clinical trials documented the efficacy of autologous PRP injection in combination with 308 nm excimer laser, fractional carbon dioxide laser or NB-UVB therapies [199,200,201]. However, none of these clinical trials investigated reparative mechanisms for oxidative stress. Despite the proven effectiveness of PRP for vitiligo repigmentation, literature evidence suggests the necessity for more accurate standardization of processing protocols. PRP isolation is not without risks, as suboptimal preparation may include harmful factors. Specifically, erythrocyte contamination during collection can induce localized inflammation at the graft site, driven by the high concentration of ROS associated with these cells [202]. Lastly, the intrinsic oxidative stress documented in vitiligo may limit the reparative character and the utility of autologous materials.

**Table 2:** Therapeutic efficacy of enzymatic and non-enzymatic antioxidants in vitiligo clinical trials.

Agent	Mechanism of Action	Study Type/Key Findings	References
Vitamin E	↓UV-induced lipid peroxidation; ↑melanocyte proliferation; redox and immune modulation.	**Randomized Controlled Trial:** Enhanced efficacy of corticosteroids, calcineurin inhibitors, vitamin D analogs and phototherapy	[159,160]
Vitamin C	ROS neutralization; ↓oxidative damage; slows melanocyte degeneration.	**Prospective/Trial:** Effective stress mitigation when combined with NB-UVB or topical steroids.	[159,161]
Zinc	Anti-apoptotic and antioxidant; modulates melanogenesis and cell-mediated immunity.	**Clinical Trials:** Synergy with steroids/antioxidants + 308-nm excimer for superior repigmentation	[163,164,165]
Copper	↑Melanogenesis; protection against oxidative stress	**Randomized Prospective:** Significant repigmentation when combined with antioxidants and excimer	[153,165]
Selenium	Essential for glutathione peroxidase activity; ↓free radical damage	**Clinical Observation:** Supportive of repigmentation when administered within antioxidant complexes.	[165]
Alpha-lipoic acid (ALA) and Polyunsaturated fatty acids (PUFA)	↑Melanocyte replication; regulates immunosuppression and redox balance	**Clinical Trials:** Significant repigmentation enhancement with NB-UVB or betamethasone	[109,159,167]
Catalase plus superoxide dismutase Gel	Restoration of the redox balance degrading O_2_^−^ to H_2_O_2_ (SOD) and this latter in H_2_O and O_2_ (CAT)	**Clinical Trial:** Combination with NB-UVB promotes significant repigmentation.	[151]
Pseudocatalase	Complex capable of degradation of H_2_O_2_ to O_2_ and H_2_O after photo-activation with UVB or solar irradiation	**Randomized Controlled Trial:** Efficacious with UVB; however, no incremental benefit over NB-UVB alone in some trials	[150,152]
Pseudocatalase/ superoxide dismutase	Restoration of the redox homeostasis	**Randomized Controlled Trial:** No clinical superiority over tacrolimus monotherapy when used in combination	[154]
Superoxide dismutase	Superoxide anion dismutation; ↓ downstream inflammatory mediators	**Randomized Controlled Trial:** Significant repigmentation synergy with NB-UVB or 308-nm excimer	[153,155]
Coenzyme Q10	Lipophilic antioxidant; regenerates other antioxidants; ↓UVA/H2O2 oxidative effects	**Randomized Controlled Trial (Placebo-controlled):** Significant decrease in VASI score after 8 weeks of treatment	[156]
Ginkgo Biloba	Free radical scavenging; ↓lipid peroxidation; ↑melanogenic pathways	**Clinical Trials:** Effective as monotherapy in arresting depigmentation progression	[170,171,172]
*P. emblica*	Anti-inflammatory, immunomodulatory, potent antioxidant	**Comparative:** Increased efficacy of phototherapy when combined with Vit E and carotenoids	[160]
Polypodium leucotomos	Photoprotective plant extract; antioxidant capabilities	**Randomized Controlled Trial:** Enhanced repigmentation when paired with PUVA or NB-UVB	[173,174,176]
Nigella sativa	↓ROS generation; shields against oxidative harm.	**Clinical Study:** Significant repigmentation (hands, face, and genitals) with monotherapy cream.	[175]
Licorice (Glycyrrhiza glabra)	Antioxidant and anti-inflammatory properties	**Clinical Study:** Superior efficacy in repigmentation when added to conventional therapy	[181]
Turmeric (Curcuma longa)	Free radical quenching; anti-inflammatory	**Randomized Controlled Trial:** Slightly superior efficacy of tetrahydrocurcuminoid cream + NB-UVB vs. monotherapy	[178,179]
PRP	↑Melanocyte regeneration and adhesion; anti-inflammatory effects¸ ↑antioxidant capabilities	**Randomized Controlled Trial:** Enhances repigmentation with Excimer/CO2 laser; oxidative mechanisms still uninvestigated	[194,195,196,197,198,199,200,201,202]

## 10. Conclusions

Oxidative stress is an important factor in the destruction of melanocytes, which are particularly susceptible to it, because melanin’s formation already produces ROS as a by-product. So, ROS play a crucial role in the initiation of vitiligo, particularly in genetically predisposed individuals. In fact, the presence of allelic variants or different transcriptional levels of antioxidant enzymes, in addition to specific HLA polymorphisms, seem to be responsible for alterations in regulatory mechanisms of oxidative response. ROS overload in vitiligo patients seems to produce the dysfunctions of intracellular organelles, including aberrant mitochondrial activity, ER stress and peroxisomal defects, leading to metabolism abnormality, lipid peroxidation, adhesion defects, premature cell senescence and melanocyte destruction. This oxidative stress favoring a proinflammatory and immunogenic environment contributes to the pathogenesis of vitiligo. In fact, the presence of autoantigens from stressed melanocytes and production of cytokines and chemokines triggers innate and adaptive immunity. CD8+ CTLs kill melanocytes, while Treg cells fail to suppress autoreactive immunity. In addition, biochemical and functional alterations of vitiligo hit also peripheral blood cells, proving a systemic metabolic impairment. Since vitiligo patients have a greater susceptibility to oxidative stress, anti-ROS treatments have shown potential as therapy for preventing melanocyte degeneration and allow for long-lasting repigmentation, if in combination with standard pharmacology or phototherapy. Not only are vitamin E, C, oligoelements, fatty acids or probiotics and prebiotics integrated in the diet but also plant-derived compounds are emerging as potential adjuncts to clinical therapies for the management of oxidative stress in vitiligo.

Current therapeutic strategies for vitiligo focus on protecting existing melanocytes from further damage and restoring lost melanocytes to achieve effective repigmentation. But they have a limited efficacy, so new more targeted therapies, like cell-based and cell-free therapies targeting immune dysregulation, oxidative stress and melanocyte regeneration, are necessary for better management of the disease.

Our review provides the latest information on the potential role of oxidative stress in the pathogenesis and antioxidant-based supportive therapies in the treatment of vitiligo, while maintaining the idea that further research on this aspect is necessary for the development of more effective treatment strategies and for a clear understanding of the pathogenic process of the disease.

## Figures and Tables

**Table 1:** Summary of the pathological mechanisms sustained by oxidative stress involved in vitiligo onset and progression.

Molecular Mechanisms Influenced by Oxidative Stress	Contribution to Vitiligo Pathogenesis	References
**Antioxidant Deficiency** **Catalase and SOD Activity**	– Impaired enzymatic scavenging capacity, accumulation of H_2_O_2_	[12,13,14,15,16,38,39,42,43,44,49,51]
**Mitochondrial function and bioenergetics**	– Aberrant mitochondrial activity and metabolic impairment – Sustained proinflammatory environment – Feed-forward effect on ROS production	[15,77,78,79,83,84,85,86]
**ER stress and UPR response following accumulation of oxidized proteins**	– Neo-antigens originated from melanocytes’ misfolded protein triggering autoimmunity – Fostering expression of pro-inflammatory cytokines/chemokines and T-cells homing	[98,101,103,104,105,107,108,109,110]
**Lipid peroxidation**	– Impairment of integrity and functionality of cellular membranes and intracellular signaling	[15,52,77]
**DNA damage**	– Promoting cell senescence and cell death	[13,72,118]
**Cell adhesion**	– Protein oxidation and lipid peroxidation interfere with adhesion molecules on melanocyte surface promoting anoikis	[115,116]
**Stress-induced senescence**	– SASP phenotype	[13,120,121,122]
**Inflammasome Activation** **NLRP3/IL-1β**	– ROS-mediated activation of the NLRP3 inflammasome converts oxidative stress into a chronic inflammatory signal	[118,138]
**Activation of immune system**	– Promoting inflammation and DC/CTL activation – Increased expression of CXCL9/10 via HIF1α activation resulting in lymphocytes recruitment – Increased HSP70i expression that favors melanocyte antigens presentation on MHC	[123,124,129,131,138,139,140,141,143,144,145,146,147,148]

## Data Availability

No new data were created or analyzed in this study. Data sharing is not applicable to this article.
